# Coronavirus and domestic violence: Practices for dealing with a double emergency

**DOI:** 10.1177/1473325020981091

**Published:** 2021-03

**Authors:** Albertina Pretto

**Affiliations:** Department of Law, Economics and Sociology, The Magna Graecia University of Catanzaro, Catanzaro, Italy

**Keywords:** Coronavirus, domestic violence, social work practice

## Abstract

This short essay aims to reflect on an unexpected effect of the Coronavirus in Italy: the increase of domestic violence. Through some data and qualitative interviews gathered with social workers of anti-violence centres, the essay presents the ways in which this emergency has been faced during the Coronavirus outbreak and the importance of spreading and maintaining new practices in this area for the future.

Although the phenomenon of violence against women has long existed in history, it is only since the 1990s that the United Nations Committee on the Elimination of Discrimination Against Women (UN-CEDAW, 1992) introduced the concept of gender-based violence, which includes all forms of violence against women as a female gender. This concept is confirmed in the Declaration on the Elimination of Violence against Women in which the phenomenon is defined as ‘any act of gender-based violence that results in, or is likely to result in, physical, sexual or psychological harm or suffering to women, including threats of such acts, coercion or arbitrary deprivation of liberty, whether occurring in public or in private life’ (UN, 1993: 2) and as ‘a manifestation of historically unequal power relations between men and women, which have led to the domination over and discrimination against women by men’ (UN, 1993: 1). It is therefore stated that inequality is not only a consequence of violence, but also constitutes its foundation. The Declaration also specifies that the different forms of violence against women effectively are a violation of human rights and fundamental freedoms: this recognition represents a cornerstone in international rights, and is of particular importance considering that violence against women is the most widespread and persistent violation of human rights in the world (Loi et al., 2019).

While judicial data based on the victims' denunciations largely underestimate the extent of the phenomenon, surveys carried out both nationally and internationally to detect the spread of the various forms of violence, not only present dramatic numbers but reveal that the phenomenon is global and affects women of all ages, educational levels, ethnicities, religions and socio-economic backgrounds (Agnello Hornby and Calloni, 2013). Surveys also show that violence against women is mostly prevalent in domestic environments. The Council of Europe (2011: 3) defines domestic violence as ‘all acts of physical, sexual, psychological or economic violence that occur within the family or domestic unit or between former or current spouses or partners’. Italian data – very similar to the international (WHO, LSHTM and SAMRC, 2013) – show that 31.5% of women have undergone at least one physical or sexual violence. In more than half of the cases it was domestic violence: in particular 13.6% of Italian women suffered physical or sexual violence from their partner or ex-partner and 2.6% by another family member. In 2014, 77% of the murders of women were committed within a relationship: 46.6% by the partner, 8.1% by the ex-partner and 22.3% by another family member (ISTAT, 2014, 2019).

To complicate this situation, in Italy and around the world, the Coronavirus (Covid-19) outbreak started. In short, while the world looked at what was happening in China, on January 30, 2020 the Italian government confirmed the virus was detected in two Chinese tourists visiting Italy. The first case of secondary virus transmission occurred in a municipality of Lombardy on February 18, 2020. In the same month, the number of infected and dead people started to grow rapidly, so much that, after a few weeks of partial restrictions, on March 9, the Italian government imposed a national lockdown. At the end of March, with about 90,000 people infected and 9,000 deaths, Italy was the European country hit hardest by what, in the meantime, had been declared a pandemic by the World Health Organization. Today (September 20, 2020), the situation in Italy has certainly improved so much that the lockdown has been completely lifted and the remaining restrictions are scarce. However, with the return from the summer holidays and the reopening of schools, an increase in infections has been recorded again.

The compulsory confinement imposed by the lockdown forced Italians, as well as people of other countries, to uninterruptedly share physical and emotional spaces with family members and/or partners. If such a constraint can exacerbate the domestic atmosphere and give rise to conflicts, this happens even more likely in those relations already characterized by violence. Thus, if staying indoors was the only solution to avoid contagion, for many women (and children) this was not synonymous with safety. Furthermore, due to the lockdown, many anti-violence centres and shelters suspended or limited their information, listening and accommodation services.

Already during the weeks in which partial restrictions were imposed, the number of calls and denunciations for mistreatment and violence dropped dramatically and in the remaining anti-violence centres still open the calls requesting for help were even completely zeroed. From March 8 to 15, calls received at 1522 (free 24-hours national anti-violence and stalking phone number) decreased by 55.1% compared to the same days in 2019 (Ruggiu and Console, 2020). It should also be noted that, until then, when a woman called 1522, the operator directed her to the nearest anti-violence centre: a step that required, therefore, a few minutes of waiting and an additional call, a step that required precious time for those who are forced to hide to make a call. It's easy to be afraid to call when the man who hurts you is always there and the spaces for privacy are reduced to a minimum.

Through eleven qualitative interviews gathered (via Skype) with social workers who work to help female victims of violence, in this essay I try to describe and reflect on how this unexpected effect of Covid-19 has been faced.

The social workers of the structures that help and protect abused women promptly reported to the government the absence of requests for help, highlighting an emergency within the emergency.

Starting from this report, government, social workers and local institutions cooperated to activate new practices to deal with this situation. The government released annual funds reserved for anti-violence services without going through the usual bureaucracy and urged local institutions to support these services with innovative solutions. In a few days, the closed anti-violence centres were reopened and restarted their activities, organizing interviews via telephone or via Skype, often guaranteeing telephone availability 24-hours and also activating WhatsApp numbers. The government launched an advertising campaign on social media and national televisions to raise awareness on the 1522 number, to which the 1522 app has been added; it allows women to chat with the operators and ask for information and/or help avoiding the risk of being heard by their cohabiting offenders. This new app is equipped with geolocation and by just clicking on it the police intervene. Another app (Youpol), previously reserved for reporting bullying and drug dealing events, has been adapted to receive reports of domestic violence - even anonymous - not only by abused women but also by relatives, friends or neighbours.

But there are women who cannot phone or chat because they do not have a mobile phone or who have it but without the Internet connection: these women must go to a centre in person to seek help. To move under lockdown, a self-certification form was compulsory. This document had to report the place of destination and the reason why the person was leaving his/her home. A dangerous document for an abused woman: if the document was found by the offender, a new episode of violence would probably have been sparked. If instead she had been stopped by the police for a check, she would have had to reveal that she was going to an anti-violence centre: difficult to do it if she lived in a village where, more or less, everyone knows each other and where the policeman could also be a friend or relative of the man abusing her. Moreover, anti-violence centres, present in all cities and towns, rarely exist in villages. So, taking a cue from practices already in use in other countries, the government asked pharmacies to make forms available for the victims to fill in and then send them to the nearest anti-violence centre. This initiative is spreading throughout Italy thanks to the collaboration of the National Pharmacies Associations and the National Association of Italian Municipalities. However, it should be noted that, in Italy, there are many villages where there is neither an anti-violence centre nor a pharmacy: to make up for this shortcoming, they are thinking of equipping grocery stores with the same form.

This combination of initiatives have lead women, once again, to ask for help, and from mid-March to the end of May their calls increased by 73% compared to the same period in 2019 (ISTAT, 2020). The anti-violence centres and shelters had to find new solutions to start accommodating abused women again, since the admission of new women had to respect the health safety rules imposed by the lockdown: the new arrivals could be carriers of the virus and, therefore, new housing in which to quarantine them were needed. To make up for the lack of housing, social workers worked imaginatively according to the availability of the different territories: for example, an anti-violence centre in Veneto has rented holiday homes through Booking. Other centres have turned to convents, and owners of vacant apartments or hotels closed due to the lockdown.

Usually, in Italy, the abused women (and children) are the ones to leave their homes but, in consideration of the emergency, the prosecutor of the Province of Trento has established that in cases of domestic violence, the male abuser will have to leave. The hope is that this legal measure will be extended throughout the national territory. In particular, if we take into account a possible evolution related to the pandemic, it must be considered that the social and health situation created by the Coronavirus has been (and is) a terrifying event that can trigger post-traumatic stress disorder (PTSD) in people. In recent months, Italians have faced many stressors: the loss of personal freedom, the death of friends and family members, the fear of getting sick and dying, and the confinement in their homes that could be too small or unsuitable for a compulsory seclusion, the continuous coexistence with family members, the job loss and more. Given that outbursts of anger are a symptom of PTSD (Orth et al., 2008; Orth and Wieland, 2013), it should be considered that in the coming months there may be a further increase of domestic violence as an effect of PTSD emerging in men.

To conclude, it seems important to briefly reflect on the evolutionary path of social work in the Italian setting, leading to an evolution that has made it possible to face this double emergency in the way described. For ages, social work in Italy has undertaken a subordinated role compared to the creation and application of social policies, so that the most important decisions (targets to achieve, planning of interventions, delivery procedures of the service, etc.) were made following the indications imposed – even legally – by the Government. As it was organised so rationally and inflexibly, the social work system, however, tended to remove responsibility from social workers, transforming them into sole executers of rules, regulations and action plans (Scaglia, 2005).

Approximately 30 years ago, social work began an important transformation, thanks to the reshaping of the Government’s role in social policies and administrative decentralisation of the management of the services. This change has concretely shown its effects particularly in recent years: the assignment of administrative functions to local institutions has, in fact, also entailed the transfer of planning and operative competencies, so that at the end of the ’90s, the local bodies ended up having to undertake a propositional and planning role in providing answers to the territorial needs (Siza, 2001). As a consequence, even social workers evolved in their profession. If for a long period of time they were involved almost exclusively in the management of single cases and application of intervention procedures, recently they have begun to turn their attention also to the surrounding territory and community, and have also started to consider themselves as builders of social policies as they are experts of the local needs and resources (Perino, 2010).

Therefore, it is extremely important for social workers to be aware of how much they have been able to put into practice during the pandemic outbreak: the Coronavirus - albeit dramatically - was a stimulus for social workers to autonomously find new solutions to unexpected problems and to urge the institutions to innovate and improve aid and protection practices for female victims of violence. Each operator acted as best he/she could even in relation to the situations and resources of the territory. All this cannot be lost once the pandemic is over, and thus be considered only as a single case and not as an event from which procedures to be consolidated for future cases should be drawn. It is important to reflect on the need to maintain and consolidate these new practices even when the virus will (hopefully) be completely under control: as a matter of fact, even after the Covid-19 there will be women who will find it difficult to make a call or move around, and included are those living in villages where anti-violence centres are absent. The development of new practices – as shown by this last example – is not only useful for the solution of an immediate case but also for previously existing problems or problems we were previously unaware of.

Finally, since the double emergency of Coronavirus and domestic violence are global phenomena, it would be useful to make these improved practices (which can be further developed) known. It would be equally important that, practices started up by other countries be diffused in order to constitute a set of good practices which can be implemented in other territorial contexts in which the pandemic is still ongoing, and for other types of emergencies that could occur in the future.
